# The Value of Sentinel Node Biopsy in Patients With Higher-Risk (T3, T4) Primary Cutaneous Melanomas: Insights Provided by a Stage II Risk Calculator

**DOI:** 10.1245/s10434-024-16699-3

**Published:** 2025-05-02

**Authors:** Thomas E. Pennington, John F. Thompson, Robyn P. M. Saw, David J. Coker, Serigne N. Lo, Alex H. R. Varey

**Affiliations:** 1 Faculty of Medicine and Health, The University of Sydney, Camperdown, Australia; 2 Melanoma Institute Australia, Wollstonecraft, Australia; 3 Department of Melanoma and Surgical Oncology, Royal Prince Alfred Hospital, Camperdown, Australia; 4 Department of Plastic and Reconstructive Surgery, Westmead Hospital, Westmead, Australia

It is well-established that patients with stage IIb and IIc melanomas have a worse prognosis than those with stage IIIa disease, and a prognosis similar to that of stage IIIb patients. Given that several prospective trials of adjuvant anti-programmed cell death protein 1 (PD1) immunotherapy in patients with resected stage III melanoma have demonstrated improvements of 30–40% in recurrence-free survival (RFS), it was postulated that adjuvant systemic treatment in this earlier-stage but higher-risk patient cohort with stage IIb and IIc disease might achieve proportionate improvements in the survival rates in this group. One such trial was the KEYNOTE-716 study, which randomized patients with resected stage IIb or IIc melanomas [T3b or T4 with a negative sentinel node biopsy (SNB)] to 12 months of adjuvant pembrolizumab or placebo. An interim analysis reported in 2022 showed an absolute risk reduction of 6% for recurrence or death in patients treated with pembrolizumab compared with placebo after a median follow-up of 14.3 months.^1^ The risk reduction increased to 9% at the second analysis after a median follow-up of 20.9 months. However, this advantage was achieved with a fourfold increase in serious adverse events (SAEs) in the treatment arm compared with placebo (16% grade 3/4 SAEs in the pembrolizumab group versus 4% in the placebo group).

The improved outcomes for patients with stage IIIb and stage IIIc melanoma achieved with adjuvant immunotherapy have led some to question the value of SNB for patients with clinical stage II melanoma. Indeed, there has been an increasing trend in adjuvant trial design towards omission of negative SNB status as an inclusion criterion. This is despite the clear evidence from a number of studies demonstrating the worth of SNB as a prognostic and therapeutic tool in a subset of melanoma patients.^2,3^

With these considerations in mind, we conducted a theoretical assessment of RFS and overall survival (OS) risk in patients with clinical stage IIb/c melanoma with and without the added prognostic precision provided by SNB. This was made possible by using a risk calculator for patients with stage II melanoma^4^ that was developed by Melanoma Institute Australia, which is now freely available (www.melanomarisk.org.au). This risk calculator allows assessment of recurrence risk with a negative SNB or no SNB at all and allows comparison of the risk of recurrence in patients with a SNB (and subsequent active surveillance) with the risk in patients with clinical stage IIb/c melanoma not undergoing SNB but receiving adjuvant immunotherapy.

A standardized set of patient characteristics was entered into the MIA stage II risk calculator: male, aged 50 years, with a superficial spreading melanoma of varying thickness/ulceration status (T3a-T4b) on the trunk, mitotic rate 1–5/mm^2^, no lymphovascular invasion, unknown TILs and regression, and SN status unknown or negative for each T category. This allowed analysis of outcomes across all clinical or pathological stage II disease states (IIA, B, and C). For simplicity, Breslow thicknesses of 2.1 mm and 4.1 mm were selected for T3 and T4 lesions, respectively. These patient characteristics were chosen to reflect a commonly encountered clinicopathological scenario in our practice, in an age group that has overall relatively small competing survival risks. Ideally, Breslow thicknesses in the “middle” of each T category would have been chosen. However, only T3 has an upper limit (Breslow thickness 4.0), therefore choosing a “middle” thickness for T4 is arbitrary. Consequently, and for consistency, Breslow thicknesses at the lower end of T3 and T4 were chosen. The 5-year and 10-year RFS and OS estimates were recorded for each combination, and the relative risk reductions (RRR) were calculated by dividing the absolute risk reduction by the event risk in the SNB unknown patient. Results are presented in Tables 1 and 2.

**Table 1:** Recurrence-free (RFS) and overall survival (OS) by locoregional stage (AJCC 8th edition)

T category	5-year RFS (%)	10-year RFS (%)	5-year OS (%)	10-year OS (%)
T3aN0	66	51	80	64
T3aNx	45	28	70	50
T3bN0	60	43	75	57
T3bNx	37	20	64	41
T4aN0	61	45	76	58
T4aNx	39	22	64	42
T4bN0	54	37	70	50
T4bNx	31	15	57	33

*RFS* relapse-free survival, *OS* overall survival, *N0* negative sentinel node biopsy, *Nx* sentinel node status unknown

**Table 2:** Relative risk reduction of negative sentinel node biopsy for each T-category

T category	RRR (recurrence) 5-years (%)	RRR (recurrence) 10-years (%)	RRR (death) 5- years (%)	RRR (death) 10- years (%)
T3aN0	38	32	33	28
T3aNx				
T3bN0	37	29	31	27
T3bNx				
T4aN0	36	29	33	28
T4aNx				
T4bN0	33	26	30	25
T4bNx				

*RRR* relative risk reduction

Additionally, the effect of changing the variables required by the stage II calculator on the magnitude of the risk reduction of a negative sentinel node biopsy was examined. Results are presented in Tables 3 (RFS and OS data) and 4 (relative risk reduction compared by variance of each prognostic factor). Across all categories of variables, a negative sentinel node biopsy conferred improvements in 5- and 10-year RFS and OS.

**Table 3:** RFS and OS by clinicopathological variable

Variable		RFS5		OS5		RFS10		OS10	
		N0 (%)	Nx (%)	N0 (%)	Nx (%)	N0 (%)	Nx (%)	N0 (%)	Nx (%)
Standard		54	31	70	57	37	15	50	33
Age (years)	20	71	52	88	82	58	35	78	67
	50								
	80	33	12	37	21	17	3	14	5
Sex	Female	64	42	78	68	48	24	61	47
	Male								
BT	2.1	60	37	75	64	43	20	57	41
	4.1								
	6	51	28	67	53	34	12	45	29
Primary site	Lower limb	51	28	70	57	34	13	50	33
	Upper limb	57	34	70	57	40	18	50	33
	Trunk								
	H N	52	29	70	57	35	13	50	33
Subtype	Nodular	60	38	75	64	44	21	57	41
	SSM								
	Acral	56	33	69	56	39	16	49	33
Mitoses	0	60	38	73	62	44	21	55	39
	1–5								
	6–10	55	31	69	56	38	15	48	31
	11+	50	26	63	49	33	12	41	25
Satellites	Unknown	59	36	70	57	42	19	50	33
	No								
LVI	Unknown	54	31	70	57	37	15	50	33
	Yes	38	16	70	57	21	5	50	33
	No	54	31	70	57	37	15	50	33
TILs	Yes	57	34	72	60	40	17	53	37
	No	39	17	69	56	22	5	44	32
	Unknown								
Regression	Yes	54	31	70	57	37	15	50	33
	No	64	43	70	57	49	25	50	33
	Unknown								

Standard features: male, age 50 years, superficial spreading melanoma, Breslow 4.1 mm, ulcerated on the trunk, 1–5 mitoses, no satellites, LVI unknown, TILs unknown, regression unknown

*RFS5* 5-year recurrence-free survival, *RFS10* 10-year recurrence-free survival, *OS5* 5-year overall survival, *OS10* 10-year overall survival, *N0* SN negative, *Nx* sentinel node biopsy not performed, *BT* Breslow thickness, *LVI* lymphovascular invasion, *TILs* tumor infiltrating lymphocytes, *HN* head and neck

**Table 4:** Relative risk reduction of negative SNB by clinicopathological variable

Variable		RRR R5 (%)	RRR R10 (%)	RRR D5 (%)	RRR D10 (%)
Standard		33	26	30	25
Age (years)	20	40	35	33	33
	50				
	80	24	14	20	9
Sex	Female	38	32	31	26
	Male				
BT	2.1	37	29	31	27
	6	32	25	30	23
Primary site	Lower limb	32	24	30	25
	Upper limb	35	27	30	25
	Trunk				
	HN	32	25	30	25
Subtype	Nodular	35	29	31	27
	SSM				
	Acral	34	27	30	24
Mitoses	0	35	29	29	26
	1–5				
	6–10	35	27	30	25
	11+	32	24	27	21
Satellites	Unknown	36	28	30	25
	No				
LVI	Yes	26	17	30	25
	No	33	26	30	25
	Unknown				
TILs	Yes	35	28	30	25
	No	27	18	30	18
	Unknown				
Regression	Yes	33	26	30	25
	No	37	32	30	25
	Unknown				

Standard features: male, age 50 years, superficial spreading melanoma, Breslow 4.1 mm, ulcerated on the trunk, 1–5 mitoses, no satellites, LVI unknown, TILs unknown, regression unknown

*R5* recurrence at 5 years, *R10* recurrence at 10 years, *D5* death at 5 years, *D10* death at 10 years, *BT* Breslow thickness, *LVI* lymphovascular invasion, *TILs* tumor infiltrating lymphocytes, *HN* head and neck, *SSM* superficial spreading melanoma

The most powerful determinant of the magnitude of RRR was age, with younger patients benefiting more from a negative SNB and older patients benefiting less than our standard theoretical patient (10-year RRR of recurrence 14%, 25%, and 35% for ages 80, 50, and 20, respectively). Female sex conferred a greater benefit of a negative SNB than male sex with respect to 5 and 10-year RFS but did not significantly impact relative risk reduction of death. Smaller reductions in RRR with thicker tumors were noted compared with thinner tumors. Neither location nor subtype of melanoma had much effect on the magnitude of RRR with a negative SNB. A small reduction in the RRR was observed with increasing mitotic rate of the primary tumor. Moderate reductions in RRRs were observed when TILs were absent and when lymphovascular invasion was present. Tumors lacking regression had slightly greater RRR of a negative SNB compared with tumors displaying regression.

From these calculations, the prognostic value of SNB was clear. In fact, a negative SNB in the highest risk T-category (T4b) conferred equivalent OS and superior RFS compared with a patient with the lowest risk T-category (T3a) not undergoing SNB (OS, RFS T4bNo 70%, 54% versus T3aNx 70%, 45%). For T-categories T3a, T3b, T4a, and T4b, a negative SNB conferred a relative risk reduction (RRR) of death at 5 years of 33%, 31%, 33%, and 30%, respectively. Similarly, there was a RRR for recurrence at 5 years of 38%, 37%, 36%, and 33% for stages T3a, T3b, T4a, and T4b, respectively, compared with no SNB. At 10 years, for categories T3a, T3b, T4a, and T4b, the RRRs for OS were 28%, 27%, 28%, and 25%, respectively, and RFS 32%, 29%, 29%, and 26%, respectively.

## Conclusions

SNB provides valuable prognostic information above and beyond that provided by primary tumor features. This is important when discussing the risk to benefit ratio with stage II patients for whom adjuvant immunotherapy is being considered. Additionally, to be considered in the discussion is the fact that SNB greatly reduces the regional disease recurrence risk.^2^ Consequently, it seems logical to conclude that omission of SNB in patients with stage IIb and IIc melanomas would be a step backward in the pursuit of effective personalized care.
